# First-Principles Study on the Magnetoelectric and Optical Properties of Novel Magnetic Semiconductor Li(Mg, Cr)P With Decoupled Spin and Charge Doping

**DOI:** 10.3389/fchem.2020.594411

**Published:** 2020-10-08

**Authors:** Ting Chen, Nan Wu, Yue Li, Yuting Cui, Shoubing Ding, Zhimin Wu

**Affiliations:** Chongqing Key Laboratory of Photoelectric Functional Materials, College of Physics and Electronic Engineering, Chongqing Normal University, Chongqing, China

**Keywords:** Cr doped LiMgP, electronic structure, ferromagnetism, optical properties, first-principles calculations, 31.15.A-, 71.15.Nc, 71.20.-b, 85.75.-d

## Abstract

The electronic structures, magnetic and optical properties of Li_1±*y*_(Mg_1−*x*_Cr_*x*_) P (*x, y* = 0.125) are calculated by using the first principles method based on density functional theory. We find that the incorporation of Cr causes the strong hybridization between Li-2s, P-2p, and Cr-3d orbitals, resulting in a spin-polarized impurity band and forming stronger Cr-P polar covalent bonds. Li(Mg_0.875_Cr_0.125_)P becomes half-metallic ferromagnetism. The properties of the doped systems can be regulated by Li off-stoichiometry. When Li is deficient, the narrower impurity band and stronger *p*-*d* orbital hybridization enhance the half-metallicity. While the half-metallicity disappears, the band gap becomes wider, and the conductivity decreases for Li excess system, but its magnetic moments increase. Comparing optical properties show that the imaginary part of dielectric and complex refractive index function and optical absorption spectrum all have a new peak in the low energy region after Cr doping, and the new peaks are significantly enhanced when Li is deficient. The absorption range of low frequency electromagnetic wave is enlarged, and the energy loss functions show obvious red-shift effect for the doped systems. The results indicate that the properties of Li(Mg,Cr)P can be controlled by Cr doping and Li off-stoichiometry independently, which will benefit potential spintronics applications.

## Introduction

Diluted magnetic semiconductors (DMS) combine the charge freedom and spin freedom of the electrons in the same matrix and have both advantages of semiconductor and magnetic materials. Although a lot of studies have been carried out for the traditional DMS (Wolf et al., 2001; Zutic et al., 2004; Jungwirth et al., 2006; Dietl, 2010), there are still some obstacles that need to be solved. Firstly, for II-V-based diluted magnetic semiconductors, only local magnetic moments are introduced by replacing equivalent metal ions with Mn. The antiferromagnetic super-exchange interaction between local magnetic moments makes them have different magnetic behaviors at different magnetic ion concentration and temperature. Secondly, for III-V-based diluted magnetic semiconductors, due to the non-equivalent substitution of doping, the solubility of the magnetic ions is limited and only metastable film materials can be formed (Ohno, 1998), which results in the specimens only available as thin films and sensitive to preparation methods and annealing treatments. The coupled spin and charge is an obstacle not only for fundamental understanding of ferromagnetic mechanism but also for effective improvement of controllable Curie temperature (Potashnik et al., 2001; Han et al., 2019).

These challenges have attracted great attention for finding a series of new generation DMS materials. Maŝek et al. (2007) and Deng et al. (2011) firstly through theory and experiment, respectively, discovered a kind of new diluted magnetic semiconductor Li(Zn,Mn)As based on I-II-V groups. In the system, the spin is introduced by injecting Mn^2+^ in the site of Zn^2+^, and the equivalent doping makes the system has higher Mn solubility. The carrier concentration can be controlled by changing the content of Li, making Li(Zn,Mn)As has higher Curie temperature (*T*_c_) than (Ga,Mn)As. Following this, Wang et al. (2014) synthesized Li_1.1_(Zn_1−x_Cr_*x*_)As, resulting in a ferromagnetic ordering below *T*_*C*_ ~218 K, nearer to the room temperature. However, one shortcoming of Mn-doped and Cr-doped LiZnAs is the using of the toxic element Arsenic. The transition metal doped LiZnP (Tao et al., 2017) and LiZnN (Cui et al., 2019) show that the magnetic moments come mainly from the TM-3d orbitals. Kacimi et al. (2014) calculated the structurel, electronic and optical properties of 96 I–II–V and I–III–IV compound semiconductors by using first-principles theory and found that LiMgP is a direct gap semiconductor with wider band gap, which is conducive to obtaining the new DMS materials with better properties through Cr doping.

In this work, the electronic structures, magnetic and optical properties of Li_1±*y*_(Mg_1−*x*_Cr_*x*_)P (*x, y* = 0, 0.125) are calculated by using the first principles method based on density functional theory. We find that the Cr doped systems exhibit half-metallic ferromagnetism. The electrical, magnetic and optical properties of Li(Mg,Cr)P can be controlled by Cr doping and Li off-stoichiometry independently, which will benefit potential spintronics applications.

## Computational Details

**Figure 1:**
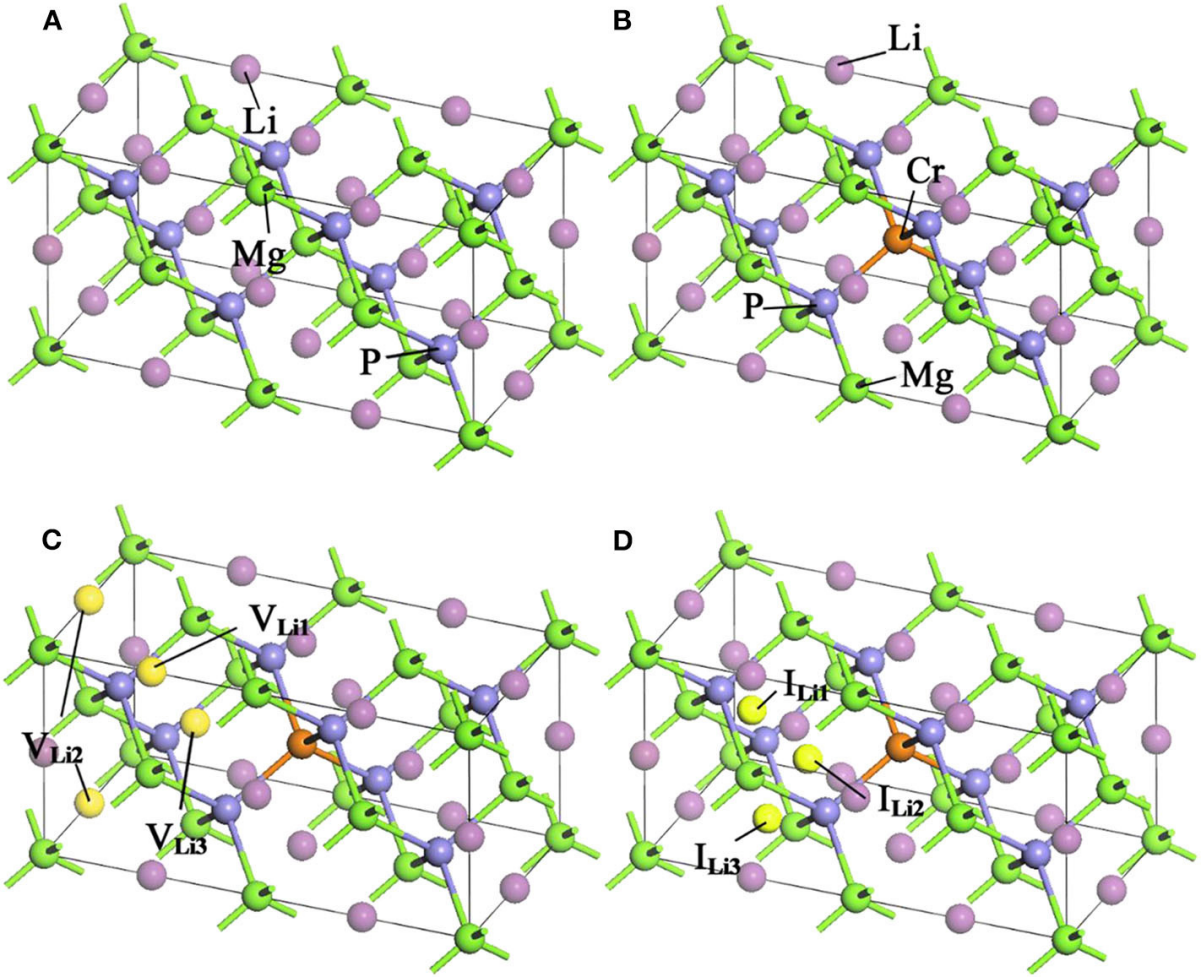
Supercell structures of Li_1±*y*_(Mg_1−*x*_Cr_*x*_)P: **(A)** LiMgP, **(B)** Li(Mg_0.875_Cr_0.125_)P, **(C)** Li_0.875_(Mg_0.875_Cr_0.125_)P, and **(D)** Li_1.125_(Mg_0.875_Cr_0.125_)P.

LiMgP is an antifluorite structure (space group *F*$\bar{4}$3 m) with the lattice constant a = b = c = 6.005 Å (Kuriyama et al., 1998). The anion P is cubic close packed, the cations Mg and Li fill the gaps in the tetrahedral, and the coordination numbers of anions and cations are 4 and 8, respectively. LiMgP can be made from high temperature solid reaction between Li, Mg, and P (Kuriyama et al., 1998). LiMgP tetrahedral lattice can be viewed as a zinc blende MgP binary compound, filled with Li atoms at tetrahedral interstitial sites near P. LiMgP is a wide-gap semiconductor with a direct forbidden band gap of 2.43 eV (Kuriyama et al., 1998). To our calculations, a 2 × 1 × 1 (24 atoms) supercell of ZB-type LiMgP is constructed containing 8 Li, 8 Mg and 8 P atoms, as shown in Figure 1A. For the doping system, the model is constructed by replacing one Mg atom in the supercell with one Cr atom, and the doping concentration is 12.5% (Figure 1B). Besides, to study the effect of Li off-stoichiometry on the properties of Li(Mg,Cr)P, the models of Li vacancy and excess systems also are constructed by removing or adding a Li atom. The different symmetry vacancy and interstitial sites (V_Li1_, V_Li2_, V_Li3_, and I _Li1_, I _Li2_, I _Li3_) have been designated in Figures 1C,D, respectively.

The first-principles calculations are carried out with the Cambridge Serial Total Energy Package (CASTEP) code based on the density functional theory (DFT) method (Payne et al., 1992; Segall et al., 2002). The periodic boundary condition is applied in all calculations, and the generalized gradient approximation (GGA) in Perdew Burke Ernzerhof (PBE) (Perdew et al., 1996) is performed to deal with the electronic exchange-correlation potential energy. In order to reduce the number of the plane wave basis vector groups, the plane-wave ultrasoft pseudo potential (USPP) method (Vanderbilt, 1990) is implemented to describe the interaction between ionic core and valence electrons. The valence electronic configurations for Li, Mg, P, and Cr are Li:2s^1^, Mg:2p^6^3s^2^, P:3s^2^3p^3^, and Cr: 3d^5^4s^1^, respectively. Single-particle Kohn-sham wave functions are expanded using the plane-wave with a cut-off energy of 400 eV. Sampling of the irreducible edge of the Brillouin zone is performed using the regular Monkhorst-Pack grid (Monkhorst and Pack, 1976) with a k-point mesh of 5 × 5 × 5. The self-consistent convergence accuracy is set at 2.0 × 10^−6^ eV/atom.

## Results and Discussion

### Structure Optimization of Li_1±*y*_(Mg_1-*x*_Cr_x_)P

To study the magnetoelectric and optical properties of novel magnetic semiconductor Li(Mg,Cr)P, the geometric structure of the original LiMgP supercell is firstly optimized by local Density Approximation (LDA) and General Gradient Approximation (GGA), respectively. The obtained lattice constant, band gaps and total energies are listed in Table 1, and compared with experimental data (Kuriyama et al., 1998). It can be found that the results obtained using the GGA reach a better agreement with the experimental data than those calculated using the LDA. As a consequence, it is more suitable to adopt GGA for the following calculations of Li(Mg,Cr)P. Besides, the geometric structures of the doping systems are also optimized with different symmetry vacancy and interstitial sites. The corresponding lattice constants, total energies and formation energies are shown in Table 2. We can find that the formation energies of V_Li1_ and I_Li1_ sites are the smallest, indicating that the doping systems of V_Li1_ and I_Li1_ sites are the most stable structures in the corresponding Li off-stoichiometry system, respectively.

**Table 1:** The calculated lattice constants, band gaps and total energies of LiMgP with LDA and GGA.

**LiMgP**	***a*(Å)**	***b*(Å)**	***c*(Å)**	**Band gap(*E*_**g**_)/eV**	**Total energy/eV**
Experiment value (Kuriyama et al., 1998)	6.005	6.005	12.010	2.43	−
LDA	5.904	5.904	11.811	1.365	−10,754.55
GGA	6.030	6.030	12.061	1.533	−10,766.57

**Table 2:** The lattice constants, total energies and formation energies of Li_1±*y*_(Mg_1−*x*_Cr_*x*_)P.

**Structure cell**	**lattice constants** **(Å)**			**Total energy(eV)**	**Formation energy(eV)**
	***a***	***b***	***c***		
Li(Mg_0.875_Cr_0.125_)P	6.005	6.005	12.010	−10,445.25	−6.569
Li_0.875_(Mg_0.875_Cr_0.125_)P-1	5.999	5.999	11.885	−12,067.47	−3.513
Li_0.875_(Mg_0.875_Cr_0.125_)P-2	5.990	6.005	11.912	−12,067.18	−3.221
Li_0.875_(Mg_0.875_Cr_0.125_)P-3	5.986	5.986	11.919	−12,067.19	−3.234
Li_1.125_(Mg_0.875_Cr_0.125_)P-1	6.038	6.0378	12.025	−12,448.96	−8.911
Li_1.125_(Mg_0.875_Cr_0.125_)P-2	6.035	6.043	12.039	−12,448.71	−8.659
Li_1.125_(Mg_0.875_Cr_0.125_)P-3	6.036	6.042	12.039	−12,448.71	−8.659

### Electronic Structure of Li_1±*y*_(Mg_1-*x*_Cr_x_)P

**Figure 2:**
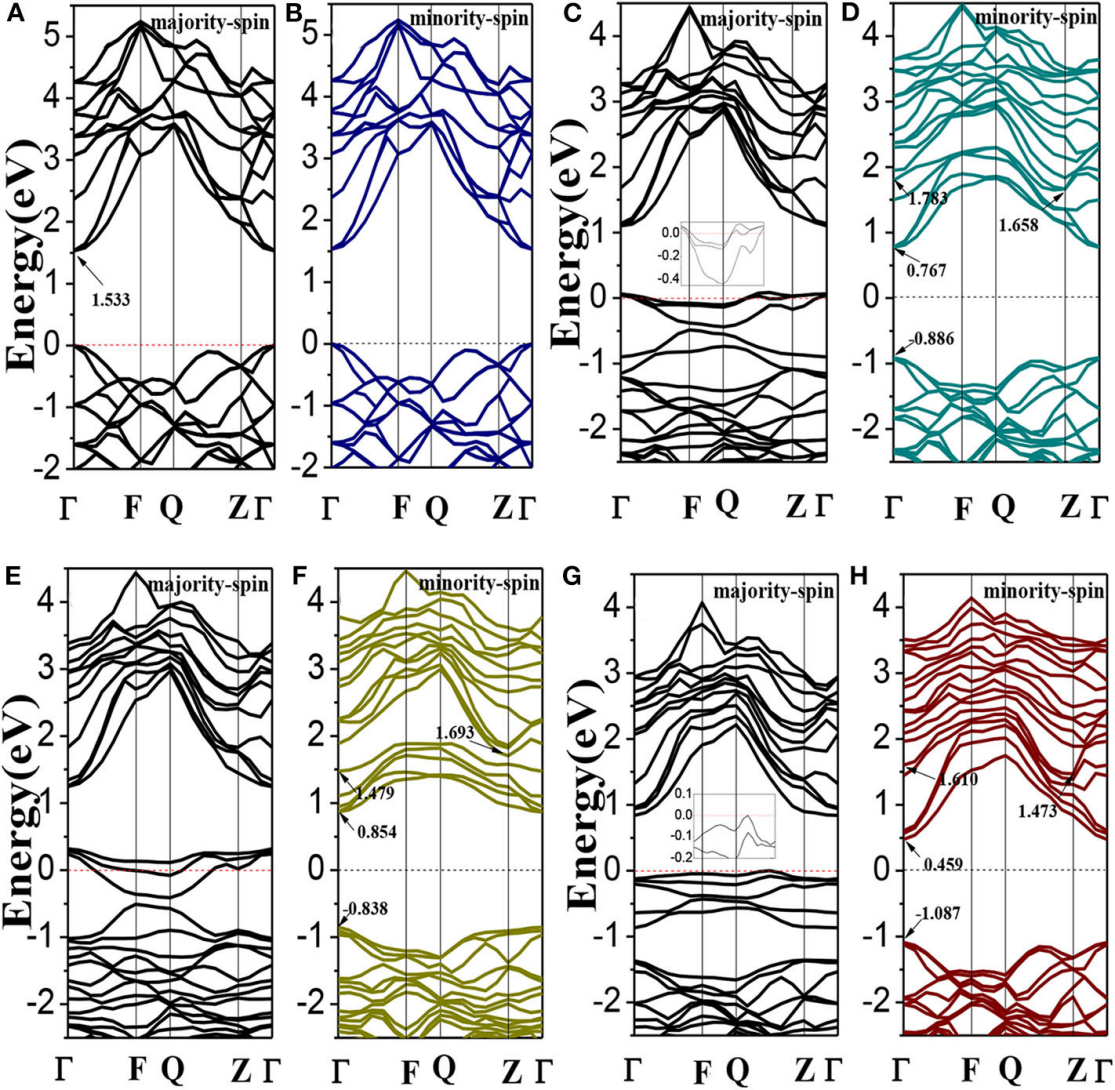
The band structures of Li_1±*y*_(Mg_1−*x*_Cr_*x*_)P: **(A,B)** LiMgP, **(C,D)** Li(Mg_0.875_Cr_0.125_)P, **(E,F)** Li_0.875_(Mg_0.875_Cr_0.125_)P, and **(G,H)** Li_1.125_(Mg_0.875_Cr_0.125_)P. Inset: enlarged views of the vicinity of the Fermi energy level.

The spin polarized band structures of Li_1±*y*_(Mg_1−*x*_Cr_*x*_)P are shown in Figure 2. The insets are enlarged views of the vicinity of the Fermi energy level. We can find in Figures 2A,B that both the valence band maxima and the conduction band minima are at the high symmetry Γ point of Brillouin-zone, indicating that LiMgP is a direct gap semiconductor. The band structures of majority-spin and minority-spin are symmetrical completely, implying that the system has no net magnetic moments. The calculated band gap for LiMgP is 1.533 eV (shown in Table 3), which is smaller than the experimental result of 2.43 eV (Kuriyama et al., 1998). This is not surprising as the underestimation of the band gap is due to the generic nature of the density functional theory (Perdew and Levy, 1983; Godby et al., 1988). Nevertheless, this has no effect on the investigation of the electronics structure and relevant properties for Cr doped LiMgP systems (Shang et al., 2004).

**Table 3:** The band gaps, impurity band widths, half-metallic gap and magnetic moments of Li_1±*y*_(Mg_1−*x*_Cr_*x*_)P.

**Li_**1±y**_(Mg_**1−x**_Cr*_***x***_*)P**	**Band gap(E_**g**_)/eV**	**Impurity band width/eV**	**half-metallic gap/eV**	**Magnetic moment/μ_**B**_**
LiMgP	1.533	–	–	0
Li(Mg_0.875_Cr_0.125_)P	2.544	1.016	0.767	4.08
Li_0.875_(Mg_0.875_Cr_0.125_)P	2.531	0.625	0.854	2.98
Li_1.125_(Mg_0.875_Cr_0.125_)P	2.560	1.151	–	4.90

As shown in Figures 2C,D, when Cr doped, the conduction band minima occur at the Γ point, while the valence band maxima are located around the Z point, in the Brillouin Zone. This result indicates that the material transforms into an indirect semiconductor. The band gap of Li(Mg_0.875_Cr_0.125_)P is 2.544 eV (Table 3), which obviously increases compared with that of LiMgP. This is because ten new spin impurity levels are emerged in band gap after doping Cr. Among them, each of the majority-spin and minority-spin bands has five impurity levels. The majority-spin impurity bands slightly cross the Femi level, demonstrating that the majority-spin bands exhibit metallic properties. While the minority-spin bands reveal still semi-conductive natures, making Li(Mg_0.875_Cr_0.125_)P system become a half-metallic material with 100% spin-polarized ratio of conduction electron. The spin-flip band gap is 0.767 eV, which are much larger than many other 3d-transition-metal-element-based materials (Guo et al., 2016). Usually, the larger spin-flip band gap, the more robust half-metallic behavior to lattice deformation and temperature (Guo et al., 2016). So the Cr doped LiMgP compounds reported in this work can be regarded as the good candidates for spintronics devices due to their large spin-flip gaps and robust half-metallicity.

Li_0.875_(Mg_0.875_Cr_0.125_)P is also an indirect band gap semiconductor with the band gap of 2.531 eV (as show in Figures 2E,F). Three majority-spin impurity bands cross the Fermi level exhibiting a metallic nature, while the minority-spin impurity bands are above the Fermi level, resulting in that the Li vacancy system also exhibits a half-metallic nature. The spin-flip band gap of Li_0.875_(Mg_0.875_Cr_0.125_)P is 0.854eV, and the half-metallicity is enhanced obviously when Li is deficient. Li excess system is also an indirect band gap semiconductor with the band gap of 2.560 eV. However, neither of the majority-spin and minority-spin impurity bands crosses the Fermi level. Therefore, Li_1.125_(Mg_0.875_Cr_0.125_)P change back to semiconducting nature again.

**Figure 3:**
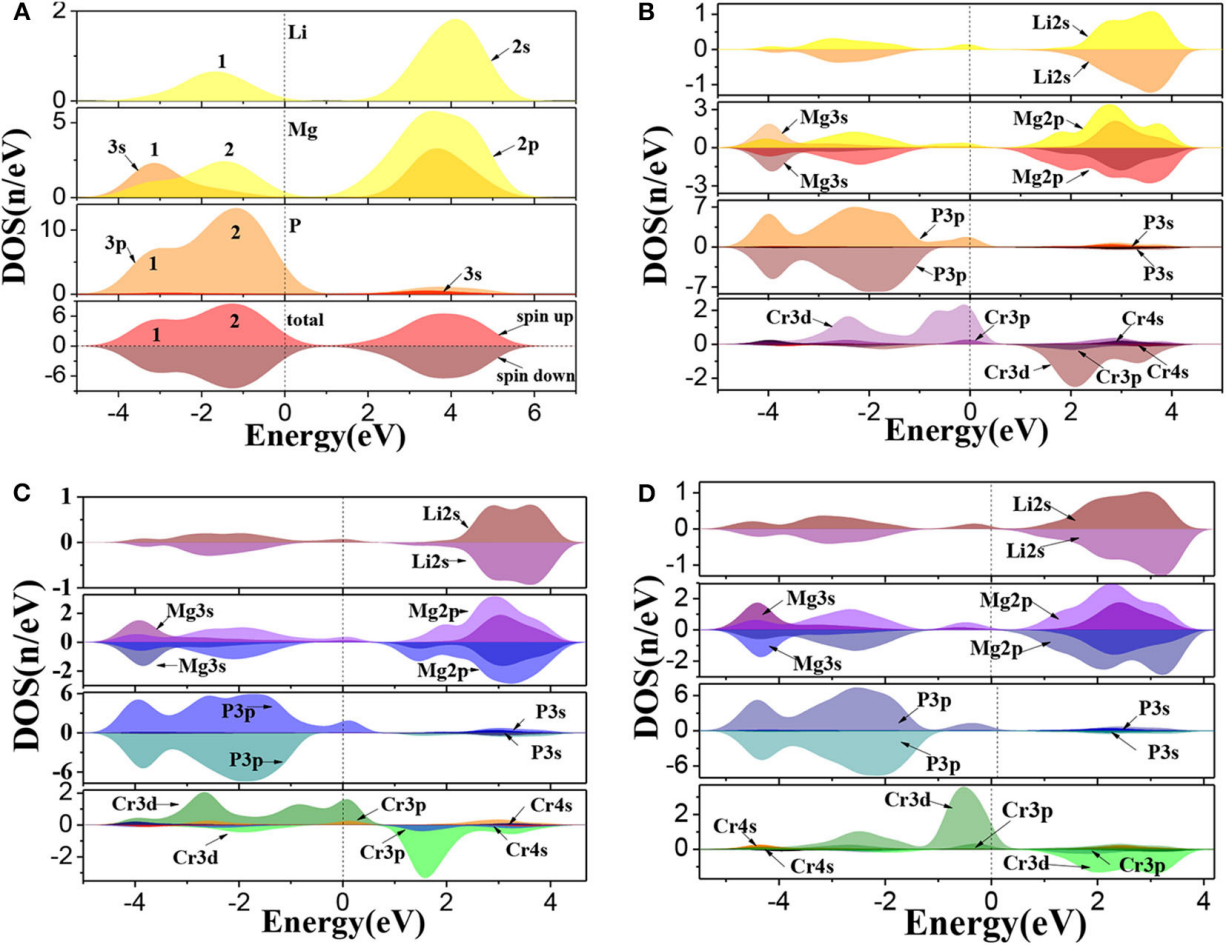
The density of states of Li_1±*y*_(Mg_1−*x*_Cr_*x*_)P: **(A)** LiMgP, **(B)** Li(Mg_0.875_Cr _0.125_)P, **(C)** Li_0.875_(Mg_0.875_Cr_0.125_)P, and **(D)** Li_1.125_(Mg_0.875_Cr_0.125_)P.

The calculated total and partial density of states (DOS) for Li_1±*y*_(Mg_1−*x*_Cr_*x*_)P are shown in Figure 3. For pure LiMgP, we can find that there are two peaks in the valence band of TDOS (1 and 2 in Figure 3A). Peak 1 is mainly contributed by the electrons of Mg-3s and P-3p states and peak 2 mainly contains the electrons of Li-2s, Mg-2p, and P-3p states. The conduction band is mainly composed of the electrons of Li-2s, Mg-2p, and Mg-3s, and the Mg-2p states. The states of spin-up and spin-down are well-symmetry, revealing that pure LiMgP has no net magnetic moment. For Cr doped system, Li-2s, P-3p, and Cr-3d orbitals hybridize near the Fermi energy (Figure 3B), resulting in that the *t*_2g_ and *e*_g_ energy levels of Cr-3*d* state are separated from each other, and the *t*_2g_ energy level is pushed toward the Fermi level, induce Li(Mg_0.875_Cr_0.125_)P to become half-metallic ferromagnetism. When Li is deficient, the electrons of P-3p and Cr-3d states have the stronger p-d orbitals hybridization (Figure 3C). The *t*_2g_ levels are pushed up even further, making its occupation states reduce from three to one, which causes that the spin-flip band gap of Li_0.875_(Mg_0.875_Cr_0.125_)P increases. When Li is excess, only a weak hybridization of p-d orbitals appears near the Fermi energy, and the *t*_2g_ levels is completely occupied by electrons. The half-metallicity disappears.

Moreover, the net magnetic moment is also calculated by integrating the occupied states below the Fermi energy and shown in Table 3. The obtained net magnetic moments are 4.08 μ_B_, 2.98 μ_B_, and 4.90 μ_B_ for the Cr doped, Li vacancy, and l Li excess system, respectively. The result indicates that the impurity band width and the net magnetic moments increase with the increasing of Li concentration.

**Figure 4:**
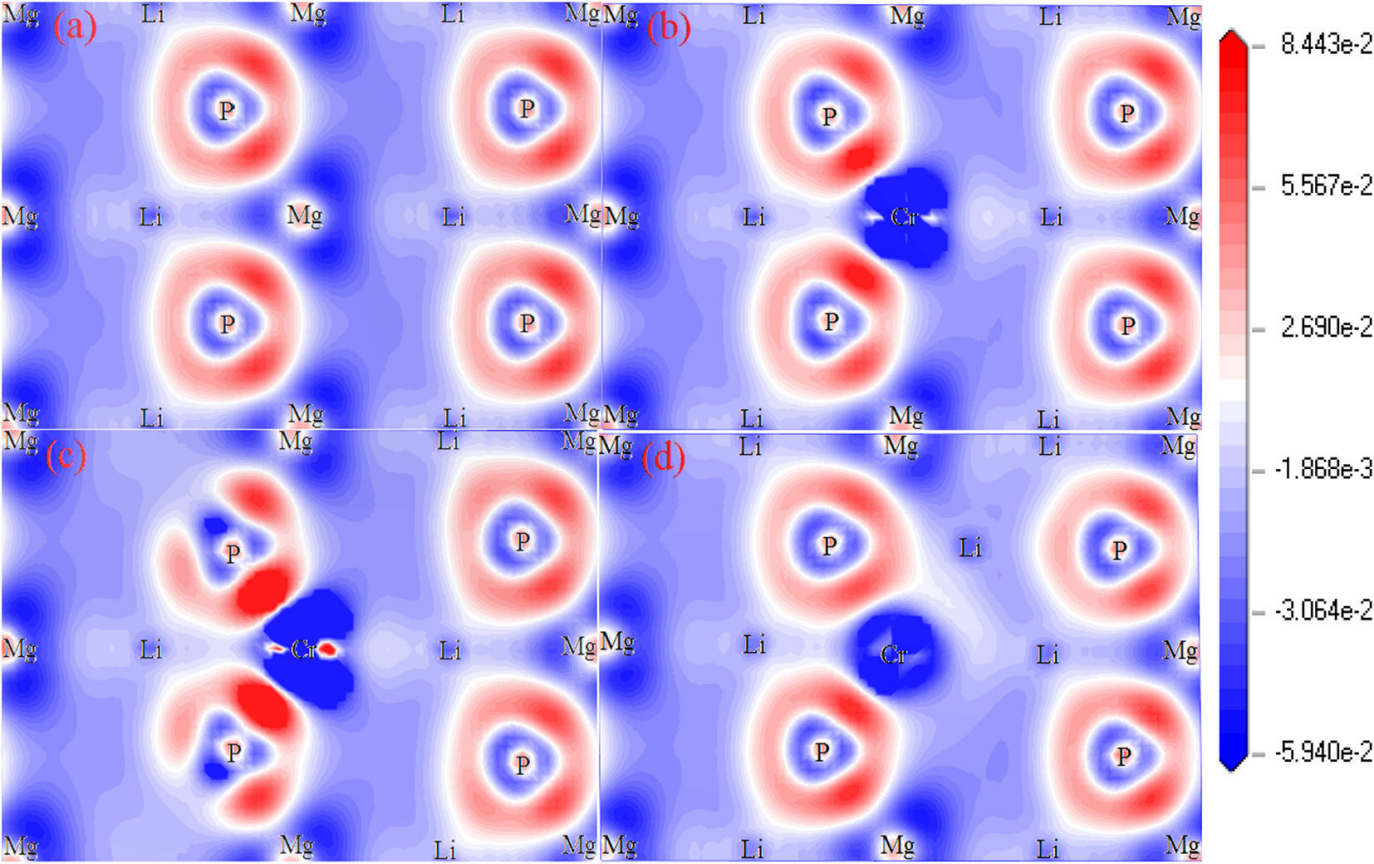
The charge density difference of Li_1±*y*_(Mg_1−*x*_Cr_*x*_)P: **(a)** LiMgP, **(b)** Li(Mg_0.875_Cr_0.125_)P, **(c)** Li_0.875_(Mg_0.875_Cr_0.125_)P, and **(d)** Li_1.125_(Mg_0.875_Cr_0.125_).

Figure 4 shows the plots of charge density difference for Li_1±*y*_(Mg_1−*x*_Cr_*x*_)P. For pure LiMgP (Figure 4a), the electron cloud of the P atom close to the Mg atom is denser. The second orbital of the P atom is polarized, and the electrons move inward. The Mg and P atoms form polarized covalent bonds. When Cr doped (Figure 4b), the P atoms gain more electrons from Cr atoms than the Mg atoms, indicating that the Cr-P polarized covalent bonds are stronger than that those of the Mg-P, which can be attributed to the strong hybridization between Li-2*s*, P-2p, and Cr-3d orbitals. When Li is deficient, the charge density between Li and P atoms becomes weaker, meanwhile, the charge density between Cr and P atoms turn stronger, indicating that P atoms cannot gain electrons from Li atoms, so the more charges need to be gained from Cr atoms. It can be seen in Figure 4d that P atoms can gain more electrons from Li atoms, resulting in that the charge loss of Cr atoms becomes less and the Cr-P polar covalent bonds become weaker for Li excess system.

### Optical Properties of Li_1±*y*_(Mg_1-*x*_Cr*_*x*_*)P

**Figure 5:**
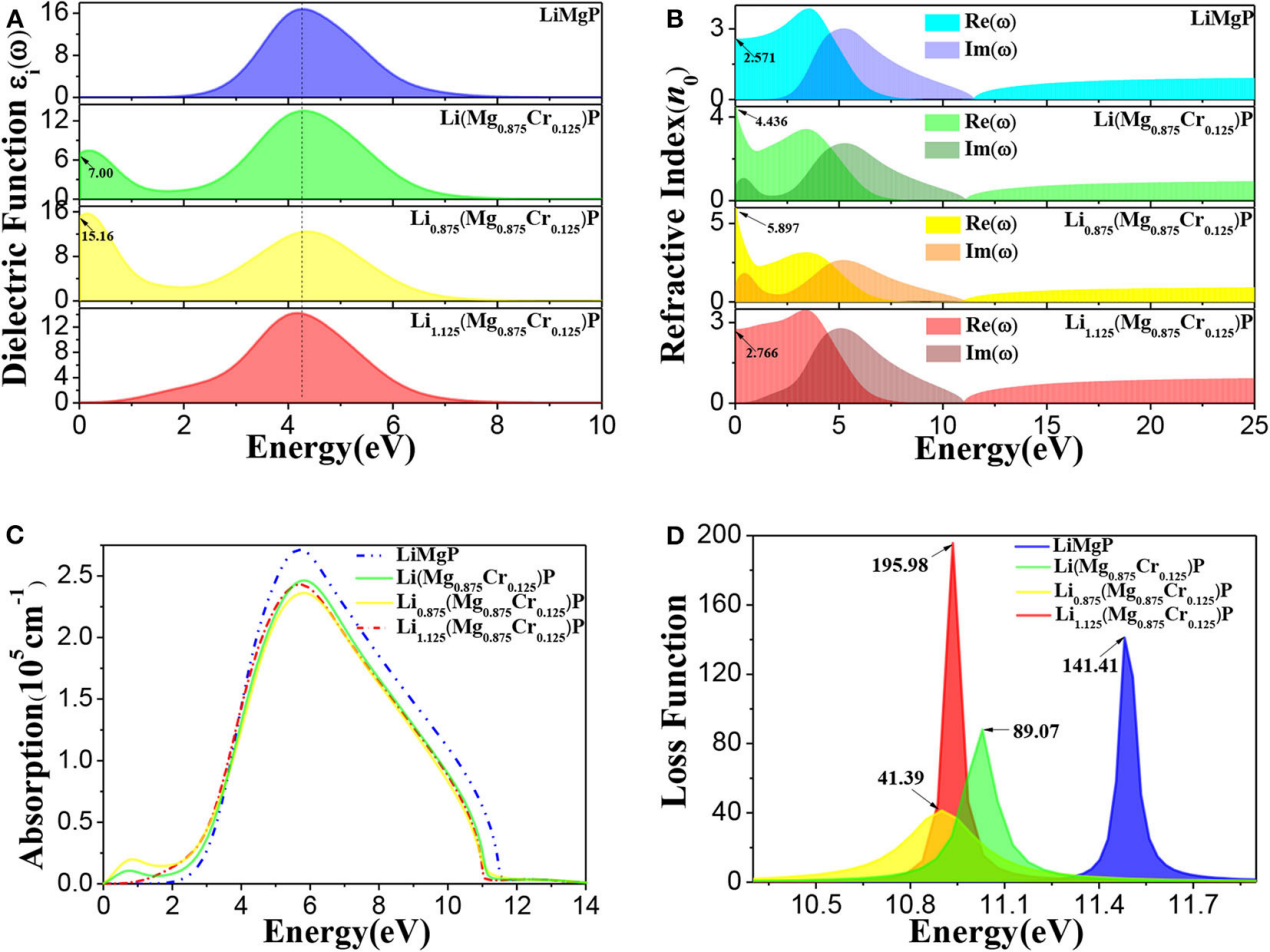
The optical properties of Li_1±*y*_(Mg_1−*x*_Cr_*x*_)P: **(A)** imaginary part of dielectric functions, **(B)** complex refractive index functions, **(C)** optical absorption spectra, **(D)** energy loss spectra.

To further investigate the effects of Cr doping and Li off-stoichiometry on properties of Li_1±*y*_(Mg_1−*x*_Cr_*x*_)P, the imaginary part of dielectric functions, complex refractive index functions, optical absorption spectra and energy loss spectra are also calculated and shown in Figure 5. We can find from Figure 5A that there is a dielectric peak at *E* = 4.27 eV, corresponding to the direct transition of P-3p in valence bands to Li-2s, Mg-3s, and Mg-2p states in conduction bands. It is worth noting that a new peak occurs in the low energy region and the peak at *E* = 4.27 eV reduces after Cr doping. Moreover, we also find that the new peak is significantly enhanced when Li is deficient, but when Li is excess the new peak disappears, and the main peak moves slightly toward the low energy region. The refractive index for pure LiMgP is 2.571 (Figure 5B). In the lower energy (*E* < 1.82 eV) and higher energy (*E* > 11.54 eV) region, the imaginary part of the complex refractive index function is zero, and the real part is a constant, indicating that the absorption of LiMgP is limit to a certain frequency range. Similarly to the dielectric functions, there are also new peaks in the low energy region of complex refractive index functions after Cr doping, implying that the absorption range of low frequency electromagnetic wave is enlarged for the doped systems. It can be seen in Figure 5C that a new peak of the optical absorption spectrum also appears in the low energy region after Cr doping, the main peak of absorption spectrum moves slightly toward the low energy region, demonstrating again that the absorption of low frequency electromagnetic wave is enhanced. As shown in Figure 5D, the energy loss functions also move obviously toward the low energy region, implying the red-shift effect for the doped systems. The energy loss for Li(Mg_0.875_Cr_0.125_)P, Li_0.875_(Mg_0.875_Cr_0.125_)P, and Li_1.125_(Mg_0.875_Cr_0.125_)P are about 63%, 29% and 138% of that for LiMgP, respectively.

## Summary

The electronic structures, magnetic and optical properties of Li_1±*y*_(Mg_1−*x*_Cr_*x*_)P (*x, y* = 0, 0.125) are calculated by using the first principles method based on density functional theory. We find that the Cr doped systems exhibit the half-metallic ferromagnetism. Due to the Cr doping, sp-d orbitals hybridization leads to spin-polarized impurity bands and form stronger Cr-P polar covalent bonds. Moreover, we also find that the properties of the doped systems can be regulated by Li off-stoichiometry. The impurity band width and the net magnetic moment increase with the increasing of Li concentration, but the half-metallicity and the conductivity decrease. When Li is excess, the p-d orbitals hybridization obviously becomes weaker and the half-metallicity disappears. Comparing optical properties shows that the imaginary part of dielectric, the complex refractive index function and the optical absorption spectrum all have a new peak in the low energy region after Cr doping, and the new peaks are significantly enhanced when Li is deficient. The absorption range of low frequency electromagnetic wave is enlarged, and the energy loss functions show obvious red-shift effect for the doped systems. The results indicate that the properties of Li(Mg,Cr)P can be controlled by Cr doping and Li off-stoichiometry independently, which will benefit potential spintronics applications.

## Data Availability Statement

The raw data supporting the conclusions of this article will be made available by the authors, without undue reservation.

## Author Contributions

All authors listed have made a substantial, direct and intellectual contribution to the work, and approved it for publication.

## Conflict of Interest

The authors declare that the research was conducted in the absence of any commercial or financial relationships that could be construed as a potential conflict of interest.
